# IGF-IR signaling in epithelial to mesenchymal transition and targeting IGF-IR therapy: overview and new insights

**DOI:** 10.1186/s12943-016-0576-5

**Published:** 2017-01-30

**Authors:** Heming Li, Izhar Singh Batth, Xiujuan Qu, Ling Xu, Na Song, Ruoyu Wang, Yunpeng Liu

**Affiliations:** 1 Department of Medical Oncology, the First Hospital of China Medical University, NO.155, North Nanjing Street, Heping District, Shenyang City, 110001 China; 2 Department of Oncology, Affiliated Zhongshan Hospital of Dalian University, Dalian, 116001 People’s Republic of China; 3 Department of Pediatrics-Research, The University of Texas MD Anderson Cancer Center, Houston, TX USA

**Keywords:** EMT, IGF-I, IGF-IR, Metastasis, Therapy

## Abstract

The insulin-like growth factor-I (IGF-I) signaling induces epithelial to mesenchymal transition (EMT) program and contributes to metastasis and drug resistance in several subtypes of tumors. In preclinical studies, targeting of the insulin-like growth factor-I receptor (IGF-IR) showed promising anti-tumor effects. Unfortunately, high expectations for anti-IGF-IR therapy encountered challenge and disappointment in numerous clinical trials. This review summarizes the regulation of EMT by IGF-I/IGF-IR signaling pathway and drug resistance mechanisms of targeting IGF-IR therapy. Most importantly, we address several factors in the regulation of IGF-I/IGF-IR-associated EMT progression that may be potential predictive biomarkers in targeted therapy.

## Background

The insulin-like growth factor-I receptor (IGF-IR) is a transmembrane tyrosine kinase receptor which regulates growth, development and metabolism by binding of the IGF-I ligands [1–3]. In recent years, mounting evidence indicates that the IGF-I/IGF-IR signaling is also involved in epithelial to mesenchymal transition (EMT)-associated tumor metastasis and drug resistance [4–9]. Overexpression of IGF-IR is associated with high risk of metastasis and poor prognosis in many cancer patients [10–14]. Therefore, IGF-IR, the key signaling component, is considered as the potential target of several investigational agents in clinical development. However, the IGF-I/IGF-IR signaling pathway seems more complex than initially thought. Failures in Phase II/III clinical trials in unselected patients prompted the scientists to pause and reevaluate the problem before conducting further trials [15–18]. In the face of these setbacks, searching for relevant biomarkers has become glaringly apparent. This review will first present EMT in tumor progression and discuss the mechanisms of IGF-I/IGF-IR signaling in regulating EMT programs in different epithelial tumor; secondly, we will consider the current strategies of anti-IGF-IR targeted therapy and analyze the reasons for treatment failure; Most importantly, we will extract candidate biomarkers and optional strategies to identify the right patients based on regulation mechanisms of IGF-I/IGF-IR-induced EMT progression.

### The key role for IGF-IR signaling in IGF system

**Fig. 1:**
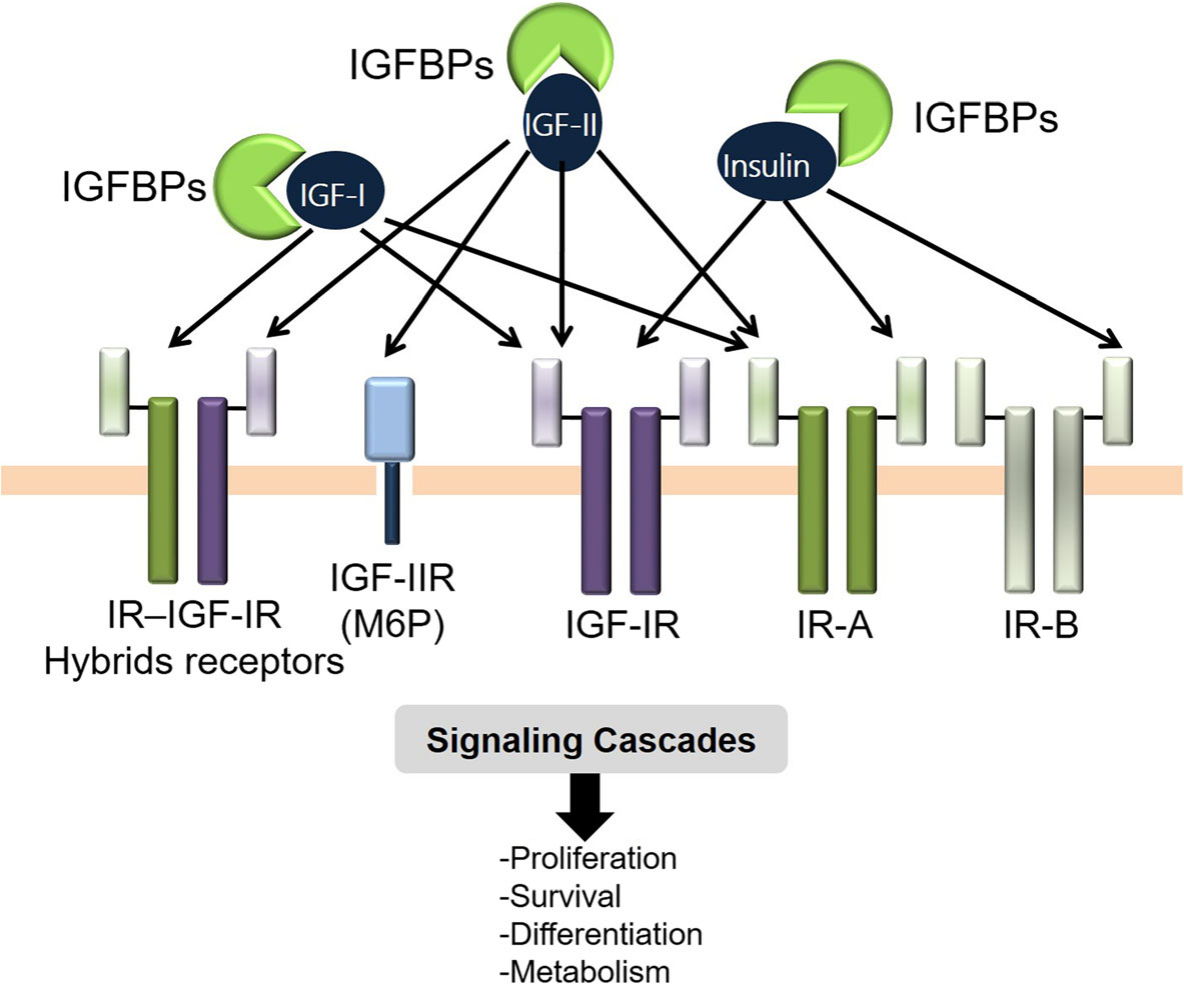
Schematic representation of the insulin and IGF receptor family. The IGF system consists of ligands (IGF-I, IGF-II and insulin), receptors (IGF-IR, IGF-II/ M6P, IR), and a family of six high-affinity IGFBPs. The IR exists in two splice variant isoforms, the IRA and IRB. Different receptors dimerize to form six receptor species those vary in their ligand affinity. Ligands binding to receptors can result in activation of different intracellular signaling cascades that regulate cell proliferation, survival, differentiation, and metabolism

The IGF system consists of three ligands: IGF-I, IGF-II and insulin; three receptors: IGF-IR, insulin receptor (IR) and IGF-IIR; and a family of six high-affinity binding proteins IGFBPs. The IR exists in two splice variant isoforms, the IRA and IRB. Different receptors dimerize to form six receptor species that vary in their ligand affinity (Fig. 1) [19, 20]. (1) IGF-I can bind to the IGF-IR, IRA, and IGF-IR/IRA receptor hybrids [21, 22]; (2) IGF-II can bind with high affinity to the IGF-IIR /mannose-6-phosphate receptor, a non-signaling receptor, which is considered to play an important role in the clearance and degradation of IGF-II [23, 24]; (3) IGF-II binds with high affinity to the IGF-IR, IRA, hybrid IGF-IR/IR receptors but not the IRB isoform [25, 26]. (4) Insulin can bind with IGF-IR and IR [1]. IGFBPs are carrier proteins that have binding affinities for both IGF-I and IGF-II. There are, at present, six members in IGFBP superfamily (IGFBP-1 through 6). IGFBPs help lengthen the half-life of circulating IGF-I due to their higher affinity to IGF ligands than the receptors. IGFBPs are also instrumental in modulating IGF-IR biological accessibility and activity [27, 28]. In biological fluids, approximately 98% of IGF-I is normally bound to one of six binding proteins IGFBPs. However, IGFBPs have a relative lower affinity with insulin [29].

The IGF-I/IGF-IR signaling is the major signal-transducing pathway in IGF family. Its activation after ligand binding mediates cell survival, proliferation, differentiation, and metabolism [30–32]. The effects of IGF-IR signaling in cancer biology are divergent. Previous studies have reported that cytoplasmic IGF-IR expression is correlated with favorable disease free survival and specific survival in estrogen receptor positive invasive ductal breast carcinoma [33]. IGF-IR expression is significantly associated with longer survival in non-small-cell lung cancer patients treated with gefitinib [34]. Whereas the opposite association is found in some other malignancies where IGF-1R exacerbated malignant transformation and tumor cell proliferation [14, 35]. This may be due to the complex and tightly regulated networks of IGF-I/IGF-IR signaling. As a potential drug target, the IGF-I/IGF-IR signaling has a number of appealing features. Many preclinical studies indicate that IGF-I induces EMT program and contributes to metastasis in breast, prostate, gastric and lung cancer [5, 36–39]. IGF-IR is involved in epidermal growth factor receptor (EGFR) TK inhibitor (TKI) resistance through crosstalk between IGF-IR and EMT signaling pathways in non-small cell lung cancer (NSCLC) with EGFR mutations [39, 40]. In addition, IGF-IR signaling mediates resistance to TKI drugs targeting both epidermal growth factor receptor 2 (HER-2) and EGFR in gastric cancer via EMT-like process [41]. In ovarian cell models, adaptive resistance to PI3K/mTOR inhibitors was associated with upregulation of IGF-IR and other pro-survival proteins [42]. Therefore, the close relationships between IGF-I/IGF-IR signaling and EMT progression makes it an attractive therapeutic target for cancer treatment.

#### EMT-an overview

EMT is a multi-step biologic process characterized by the cell-cell contacts breakdown, cell-matrix adhesion remodeling and acquisition of mesenchymal phenotype [43, 44]. EMT plays a central role in both physiological and pathological processes. It contributes to the formation of the body plan and the differentiation processes of multiple tissues and organs [43, 45]. EMT also plays as a physiological response to injury. During wound healing, keratinocytes at the border of the injury undergo EMT which maintains the loose contacts [43, 46]. As a pathological response, EMT is involved in organ degeneration, such as fibrosis [47]. Overwhelming evidence suggests that developmental of EMT program promotes the initiation of tumor metastasis and acquisition of therapeutic resistance [48, 49]. It also endows cells with stem cell properties and prevents apoptosis, which results in tumor progression [50, 51].

**Fig. 2:**
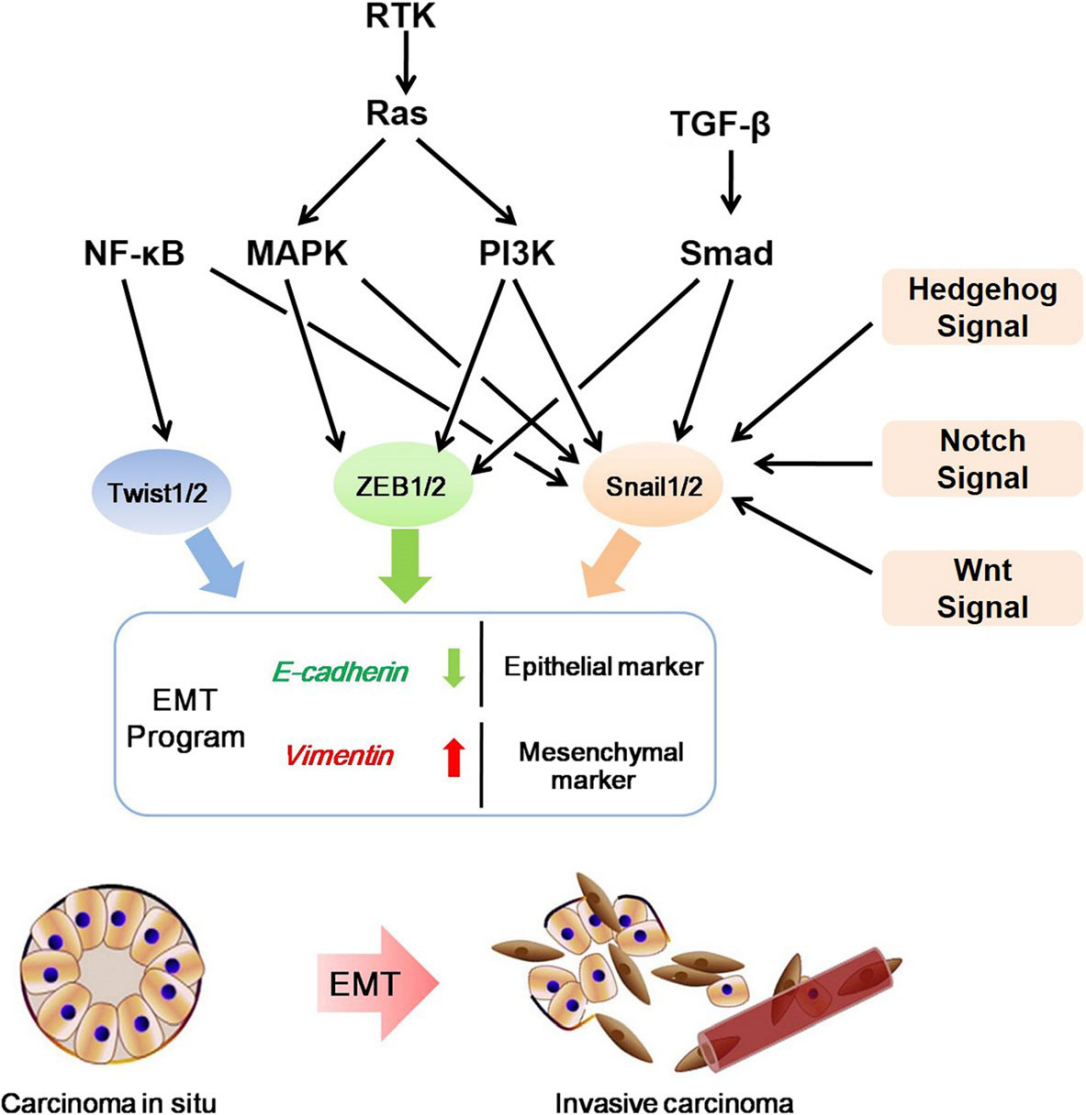
Basic molecular processes and signaling pathways contributing to the epithelial-mesenchymal transition (EMT). EMT is a developmental process by which epithelial cells lose their cell-cell adhesions and acquire mesenchymal cells identity. Loss of epithelial marker such as E-cadherin and the gain of mesenchymal marker such as Vimentin are considered as hallmarks in the initiation and execution of EMT. In many human tumors, the expression of multiple RTKs and their ligands induce autocrine growth factor loops. The activated RTKs induce signaling via PI3K/Akt and MAPK/ERK downstream signaling pathways, which up-regulates transcriptional factors (ZEB1/2 and Snail1/2) and causes EMT progression via binding to E-boxes of E-cadherin gene. TGF-β induces EMT through the activation of Smad2 signaling or other non-canonical signaling pathways (PI3K/Akt or MAPK/ERK pathways). Activation of NF-κB signaling can induce EMT program through up-regulating Twist1/2. In addition, activation of Notch, Wnt/β-catenin and Hedgehog signaling also contribute to the progression of EMT via regulation of Snail1/2

Initiating a transformation from an epithelial cell into a mesenchymal cell requires alterations in cell morphology, cellular architecture, adhesion and migration ability. Loss of the epithelial marker E-cadherin and gain of mesenchymal marker vimentin are considered as the fundamental event in EMT process [52]. Down-regulation of E-cadherin expression causes adherens junctions breakdown between cells, loss of cell polarity, leading to a mesenchymal phenotype with invasive abilities [53]. This dynamic process can be triggered by the complex interplay of several inducers, such as TGF-β, multiple receptor tyrosine kinases (RTKs), Wnt/β-catenin, Notch and Hedgehog signaling pathways [54–57]. Two important components of initiation of these complex signaling pathway networks are ZEB1/2 and Snail1/2. These EMT inducing transcription factors (EMT-TFs) can bind to E-boxes of E-cadherin promoter and repress its transcription [58–60]. Hence, any biological processes that will induce overexpression of ZEB or Snail are likely to down-regulate E-cadherin expression, which contributes to EMT. Also, some TFs supress E-cadherin transcription indirectly, such as Twist1/2, E2.2 and FoxC2 [61–63]. TGF-β induces EMT through the activation of Smad2 signaling or other non-canonical signaling pathways (PI3K/Akt or MAPK/ERK pathways) [64]. Activation of NF-κB signaling can induce EMT program through up-regulating Twist1/2 [65, 66]. Furthermore, activation of Notch, Wnt/β-catenin and Hedgehog signaling also contribute to the progression of EMT via regulation of Snail1/2 [67, 68]. These EMT-TFs not only repress E-cadherin, but also inhibit other tight junctional proteins transcriptionally, which facilitates EMT process. Additionally, newly published studies have highlighted the essential role of microRNA in the mediation of EMT process by regulating the inducers [69]. Commonly used EMT markers, inducers, pathways and transcription factors are summarized in Table 1 and Fig. 2. Nowadays, more and more studies are focusing on reinforcing EMT as a major driver factor on tumor progression, metastasis and drug resistance. Given that a complex network of regulators and inducers play integral roles in EMT, understanding the regulation mechanisms is helpful for designing more effective targeted therapies.

**Table 1:** EMT markers, inducers and transcriptional factors

EMT markers	EMT inducers and pathways	EMT-TFs
*Epithelial markers*		*Direct binding to E-cadherin promoter*
E-cadherin	RTKs (EGFR, FGFR, IGFR)	Snail1/2
Occludin	TGF-β	ZEB1/2
Desmoplakin	GSK-3β	KLF8
Cytokeratin	NK-κB	E47
Mucin1	β-catenin	Brachyury
TJP1	Hypoxia/AMF	*Repressing E-cadherin indirectly*
*Mesenchymal markers*	Ras-MAPK pathway	Twist1/2
Vimentin	PI3K/Akt pathway	FOXC2
Fibronectin	Src pathway	E2.2
N-cadherin	Notch pathway	SIX1
Thrombospondin	Wnt pathway	PRRX1
α-SMA	Shh pathway	Goosecoid
Tenascin C	mTOR pathway	HDAC
MMP family	STAT3 pathway	EZH2

*TJP1* tight junction protein 1, *α-SMA* α-smooth muscle actin, *KLF8* Krüppel-like Factor 8, *FOXC2* forkhead box C2, *PRRX1* paired-related homeobox gene 1, *HDAC* histone deacetylase

### Molecular mechanisms of IGF-IR signaling in EMT

Recently, mounting evidence indicates that the IGF-IR signaling is also involved in EMT-mediated tumor metastasis and drug resistance. The mechanism of IGF-IR signaling in regulation of EMT is summed up in three aspects: autocrine ligand production and receptor overexpression, signal transduction by ligand binding, and cross-talk between signaling pathways.

#### Autocrine ligand production and receptor overexpression

IGF-I is peptide growth factor synthesized in the liver and secreted into the bloodstream under the control of growth hormone. In the circulation, the ligands of IGF-I are combined with a family of high-affinity binding proteins (six known IGFBPs), which allows growth hormone to produce more IGF-I continuously [28, 29]. Many studies have demonstrated that slight elevations in serum levels of IGF-I are correlated with an increased risk for developing prostate, breast, colon, lung, ovarian and endometrial cancer [70–77]. Interestingly, EMT process may in turn trigger autocrine IGF-I production, thus activating a positive feedback loop between IGF-IR activation and Slug expression in vitro [78]. Furthermore, IGF-IR expression is observed in up to 80% of lung cancer patients and approaching 90% of breast cancer patients [79, 80]. Overexpression of IGF-IR promotes migratory and invasive behaviors of triple negative breast cancer cell lines by activating focal adhesion kinase signaling pathway [81]. Our newly published data has implicated that elevated IGF-IR is associated with lymph node metastasis in gastric cancer patients [37]. In the light of these discoveries, strategies that are able to inhibit the functions of IGF-IR or which are able to lower plasma levels of IGF-I should be considered with the goal of inhibiting tumor development and metastasis.

#### Signal transduction by ligand binding

**Fig. 3:**
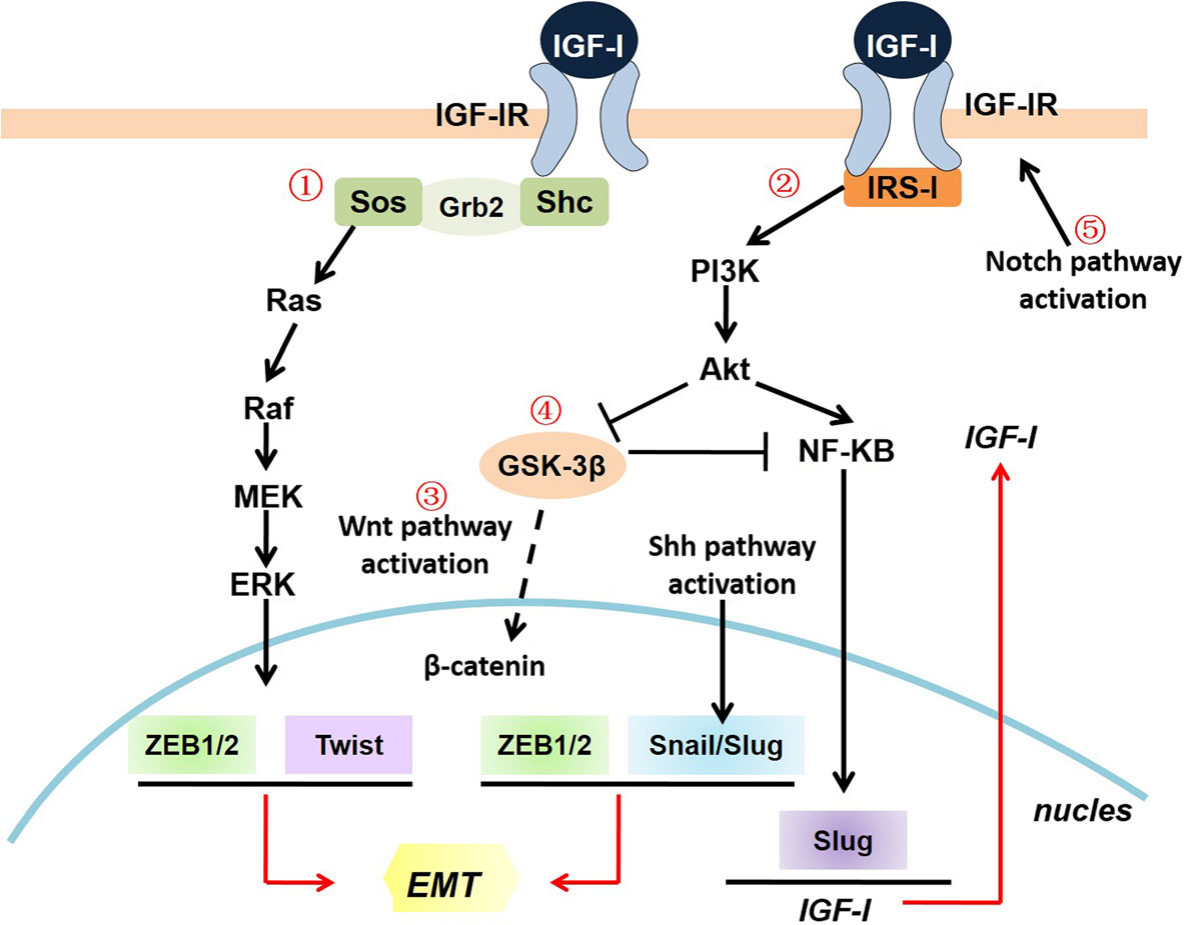
Schematic representation of IGF signaling regulation in EMT. Ligand activation of IGF-IR results in intrinsic tyrosine kinase phosphorylation and activates two main signaling pathways, ①IRS-1/PI3K/Akt and ②Ras/Raf/ERK pathways. Both of these two main pathways regulate transcription factors of ZEB, Snail and Twist families those are all involved in the EMT program. In addition, Slug increases IGF-I transcription which potentiates the progression of EMT. On the other hand, crosstalk between other signaling pathways and IGF signaling are also involved in EMT program. ③IGF-I stimulates β-catenin relocation and stability through the inactivation of GSK-3β which initiates Wnt signaling. Furthermore, IGF-I cooperates with Wnt signaling pathway in the metastasis process by stimulating TCF/LEF-dependent transcription through the Akt/GSK-3β/β-catenin pathway. ④GSK-3β binds to and phosphorylates Snail at two consensus motifs to regulate the biological functions of Snail. ⑤Notch-1 directly up-regulates IGF-IR protein and mRNA expression and amplifies the mitogenic effects of IGF-IR/PI3K signaling that potentiates EMT program.⑥Shh signaling activation mediates EMT process through up-regulation of IRS-1 and Snail

Ligand activation of IGF-IR results in intrinsic tyrosine kinase phosphorylation and activates downstream adaptor protein IRS-1 and Shc, leading to activation of two main signaling pathways, IRS-1/PI3K/Akt and Ras/Raf/ERK pathways respectively [82–84]. Activation of ERK pathway results in up-regulation of ZEB1 expression in response to IGF-I stimulation which induces EMT progression in prostate cancer [5, 85]. Our previous study demonstrated that both Akt and ERK pathways are partially involved in IGF-I-induced EMT process in gastric cancer. Inhibition of Akt/ERK pathways or knockdown of Akt/ERK gene partially reversed IGF-I-induced EMT through up-regulation of microRNA-200c which directly targets E-cadherin transcriptional repressors ZEB2 [37]. In addition to these two signaling pathways, GSK-3β is now considered as an essential EMT regulator in response to IGF-I [86]. Activation of Akt and ERK pathways result in inactivation of GSK-3β in response to paracrine/autocrine IGF-I through Ser9 phosphorylation [87, 88]. Kim et al. detected that GSK-3β was involved in direct reduction of Snail and Slug expression through proteasome-dependent degradation or NF-κB activation in response to IGF-I stimulation [89]. Zhou et al. reported that GSK-3β could bind to and phosphorylate Snail at two consensus motifs to regulate the biological functions of Snail; activation of Akt pathway led to the suppression of GSK-3β through phosphorylation of Ser9 and stabilization of Snail in response of IGF-I [90]. Our newly published data demonstrated that inhibition of Akt reversed IGF-I-induced EMT and mesenchymal phenotype in gastric cancer cells through initiating GSK-3β ability in epithelial phenotype maintenance [38]. These results indicate that the main signal transduction pathways by IGF-I ligand binding, IRS-1/Akt/GSK-3β and ERK/MAPK pathways, are potent inducers/activators in IGF-I-induced EMT process. Fig. 3 represents the relationship between the IGF-I system and the EMT process.

#### Cross-talk between signaling pathways

Several lines of evidence indicate that a strict association between the canonical Wnt/β-catenin and IGF-I signaling may contribute to EMT process [91–93]. In human colon cancer cells, IGF-I stimulates β-catenin relocation and stability through the inactivation of GSK-3β, which increases cell motility and contributes to colon cancer metastasis [94, 95]. In addition, IGF-I cooperates with Wnt signaling pathway in the metastasis process by stimulating TCF/LEF-dependent transcription through the Akt/GSK-3β/β-catenin pathway [96]. Taken together, these data indicate the existence of cross-talk and positive feed-back loop between the IGF-I signaling and Wnt/β-catenin signaling, thus contributing to cell motility and EMT process. In addition to Wnt signaling pathway, activation of Notch signaling results in up-regulation of mesenchymal markers (fibronectin, α-smooth muscle actin), down-regulation of endothelial markers (vascular endothelial-cadherin, Tie1, Tie2) and increasing migration ability in endothelial cells [97–101]. The interaction between the Notch signaling and the IGF-IR pathway has been firstly demonstrated by Eliasz et al. in lung cancer cells [102]. Notch stimulates IGF-IR transcription by regulating its promoter under hypoxic conditions. Additionally, accumulating evidence demonstrates that Notch directly up-regulates IGF-IR protein and mRNA expression [103]. The evidence of cross-talk between Notch and IGF-IR signaling represents a general mechanism that contributes to tumor progression and metastasis [104]. Another signaling pathway Shh cooperates with IGF-IR has also been reported in several cancer cells. For example, Shh signaling activation induces the up-regulation of IRS-1 and phosphorylated IGF-IR, which synergizes to promote medulloblastoma formation [105]. Furthermore, Shh signaling is also demonstrated to mediate EMT process through up-regulating Snail and down-regulating E-cadherin in NSCLC cells [106]. However, the synergistic cooperation between Shh and IGF-I signaling is not exclusive and there may be multiple sites and intermediary molecules involved in this process. A scheme depicting the cross-talk between signaling pathways in IGF-IR-mediated EMT process is shown in Fig. 3. We still need other strong evidence and validation of cross-talk mechanism involved in EMT maintenance and metastasis progression.

### Current treatment strategies-disappointment and challenges

Almost 30 candidate drugs have been tested in more than 70 clinical trials conducted in a wide variety of cancer patients through pharmaceutical, academia and biotechnology companies during the past 10 years. Novel anti-IGF-IR drugs include monoclonal antibodies, tyrosine kinase inhibitors, and anti-ligands antibodies [107–110]. However, initial high expectations quickly encountered challenges. Therapy with monoclonal antibodies (mAb) targeting the IGF-IR have been unsuccessful [111–113]. Recent PhaseIIand III clinical trials have reported the mAb targeting the IGF-IR even worsened overall survival in breast and pancreatic cancer patients [114, 115]. Two randomized phase III studies in advanced non-small cell lung cancer were closed ahead of time due to not meeting the primary endpoint of improving overall survival [116]. In addition, some serious adverse events such as pneumonia, hyperglycemia, asthenia, and dehydration are observed more commonly in patients receiving targeted IGF-IR therapy [117, 118]. For this reason, the treatment has not gained traction for clinical use.

**Fig. 4:**
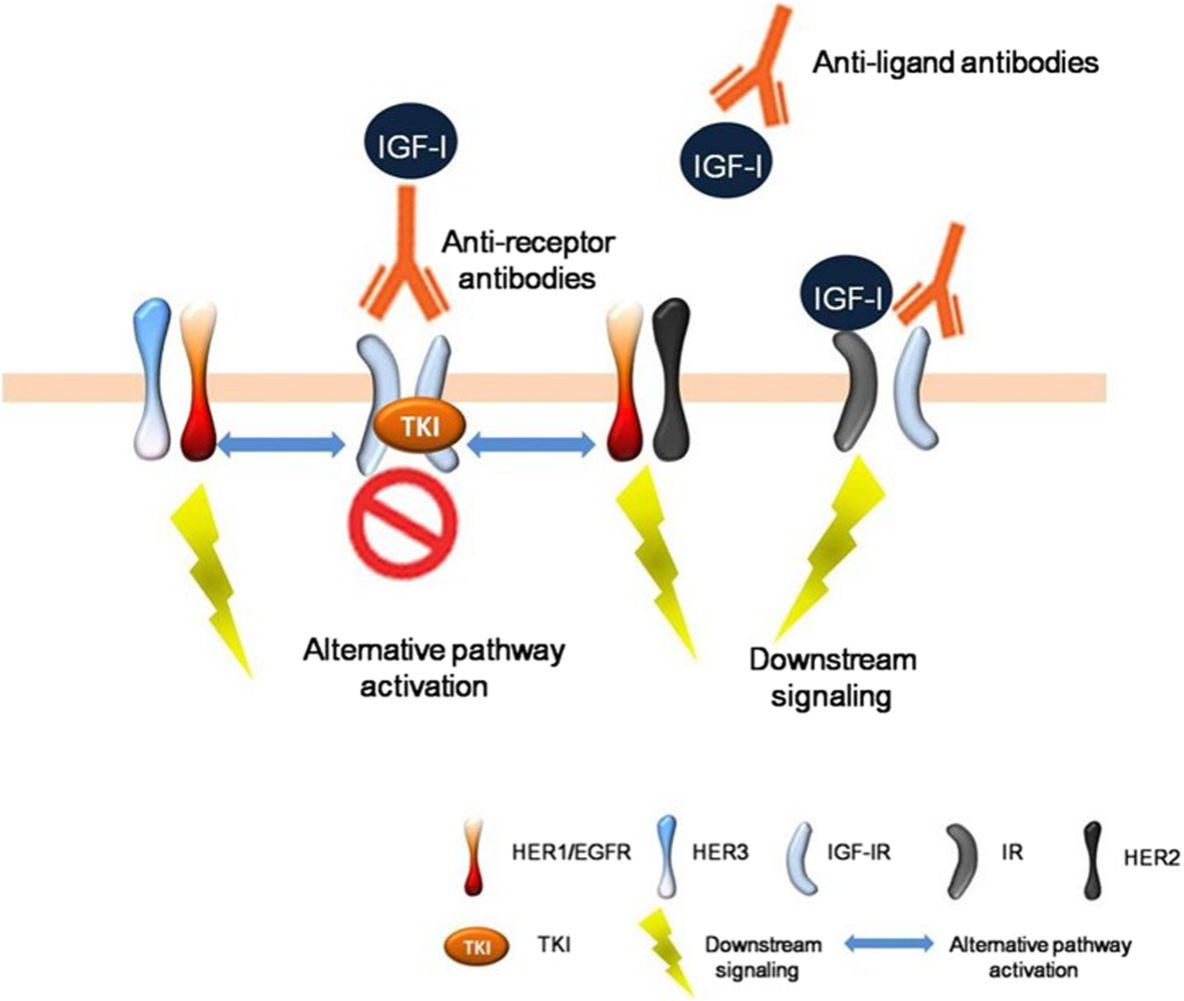
Model of IGF system inhibition strategies and resistance mechanisms. Strategies to target the IGF-I/IGF-IR axis included increasing circulating levels of IGF-I and blocking kinase activation of the IGF-IR. The mechanisms of drug resistance are mainly in abnormal autocrine or paracrine expression of ligand IGF-I, not shut down receptor signaling completely (hybrid receptor or IR signaling) or activation of alternative signaling pathway (EGFR or HER2 signaling pathways). IR, insulin receptor; TKI, tyrosine kinase inhibitor; EGFR, epidermal growth factor receptor; HER2, epidermal growth factor 2 receptor

To explain clinical failures despite encouraging preliminary data, one can consider the mechanisms of drug resistance. These include abnormal autocrine or paracrine expression of ligand IGF-I, not shut down IGF-IR signaling completely or activation of alternative signaling pathway [119, 120]. IGF-IR mAbs can induce compensatory regulatory endocrine that may lead to supraphysiological levels of IGF-I and cause increased levels of insulin in blood. Moreover, insulin receptor (IR) forms heterodimers with IGF-IR. Both IGF-I and insulin may also activate insulin or hybrid receptors and transmit intracellular signaling information even in the treatment of IGF-IR mAbs [121, 122]. High IR to IGF-IR ratios are associated with higher resistance to IGF-IR blockade [120]. Besides that, receptor tyrosine kinase reciprocity and alternative signaling pathway activation may also contribute to the IGF-IR targeting resistance. An unique interaction between HER2 and IGF-IR contributes to trastuzumab resistance in breast cancer cells [123]. Increased expression and activation of various members of HER family receptors are observed after treatment with IGF-IR/InsR inhibitor in ovarian cancer cells, suggesting that up-regulation of HER pathway is sufficient to mediate resistance to IGF-IR-targeted therapy [124, 125]. Barnes et al. reported that IGF-I stimulation would heterodimerize IGF-IR and EGFR and phosphorylate EGFR signaling pathway [126]. Intracellular feedback loops may also cause to the increased of compensatory signaling through EGFR when IGF-IR signaling pathway is targeted by mAbs (Fig. 4) [127]. Above all, it appears that the IGF-IR signaling pathway is more complex than what was initially thought to be. Overoptimistic testing in unselected patients has already yielded to such failure in IGF-IR inhibitor therapy. Therefore, careful consideration and measurement on mechanisms of IGF-I-induced tumor metastasis, finding predictive biomarkers and selecting right patients are necessary to efficiently tailor anti-IGF-IR therapy.

### Potential strategies for anti-IGF-IR therapy in cancer

#### Select right patients with predictive markers according to EMT status

**Fig. 5:**
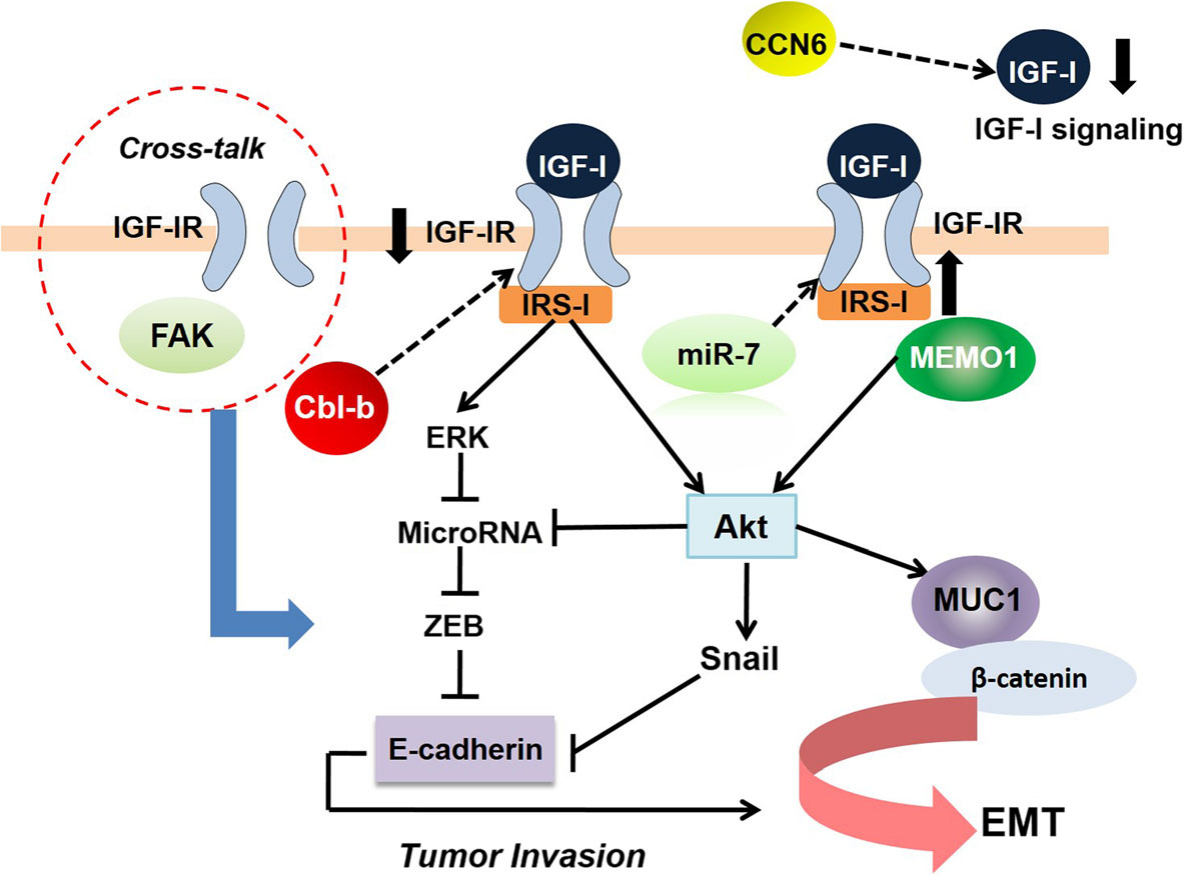
The critical factors involved in IGF-IR-mediated EMT process. IGF-IR is a transmembrane tyrosine kinase receptor. Ligand binding leads to IRS-1 phosphorylation and activate downstream PI3K/Akt and ERK/MAPK signaling pathways. An Akt-GSK-3β-ZEB2 axis and an Akt/ERK-miR-200c-ZEB2 axis exist in IGF-I-induced EMT program. Ubiquitin ligase Cbl-b targets IGF-IR for degradation and further inhibits Akt/ERK-miR-200c-ZEB2 axis in IGF-I-induced EMT. CCN6 protein contributes to the maintenance of normal breast homeostasis through decreasing IGF-I levels in the extracellular medium and repression of IGF-IR signaling pathway activation. MEMO1 triggers EMT program via the activation of the IGF-IR/IRS-1 signaling pathway. Another factor MUC1 is a critical downstream effector that mediates IGF-I-induced EMT in breast cancer cells. MicroRNA-7 reversed EMT progression through targeting IGF-IR in gastric cancer. IGF-IR/FAK crosstalk increases expression of ZEB-1 and Snail with subsequent facilitation of EMT, leading to increased cell migration and invasion in TNBC. Cbl-b, casitas B cell lymphoma-b; CCN6, WNT1-inducible-signaling pathway protein 3; MEMO1, mediator of ErbB2-driven cell motility 1; MUC1, mucin-1; microRNA-7, miR-7; FAK, focal adhesion kinase; TNBC, triple negative breast cancer

Most early clinical trials often consider serum IGF-I levels, IGF-IR or IR expression levels as the markers to predict response to IGF-IR blockade treatment [17, 114]. However, some clinical studies conclude that IGF-IR expression is necessary but not sufficient to predict the response [128–131]. In a clinical trial of IGF-IR inhibitor in osteosarcoma therapy, all of the IGF-IR mRNA expression, copy number, cell surface protein expression and gene mutation status were not associated with responsiveness to IGF-IR inhibition therapy [132]. Additionally, researchers could not find any correlations between levels of IGF-I and treatment effect to IGF-IR blockade in a negative phase 3 clinical trial for metastatic adenocarcinoma of pancreas [133]. Therefore, more effective biomarkers outside serum IGF-I level and tissue IGF-IR expression need to be utilized in fundamental research and clinical setting. Some researchers investigated whether EMT process could influence the response to IGF-IR blockade in cancers. Indeed, EMT could predict sensitivity to a dual IGF-IR/IR inhibitor OSI-906 in the hepatocellular carcinoma cell lines [8]. The combination of erlotinib (EGFR-TKI) and OSI-906 predicted synergistic inhibition of cell proliferation for hepatocellular carcinoma cells with epithelial phenotype. A subsequent molecular analysis of a negative randomized phase II/III clinical trial identified that mesenchymal phenotype was associated with dalotuzumab (a recombinant humanized mAb targeted against IGF-IR) therapy response. Hence, EMT status may be used to select those patients who are most likely to benefit from this treatment [134]. Recently, we discovered a potential biomarker for identifying lower risk of gastric cancer patients in IGF-I-induced EMT: Cbl-b [37]. Cbl-b is the second member of the E3 ubiquitin ligase Cbl family [135, 136]. Previous studies implicate that Cbl-b regulates cancer cell proliferation, drug sensitivity, and migration [137–139]. A negative correlation between Cbl-b and IGF-IR-associated tumor metastasis was recently verified [37]. Hence, patients with lower Cbl-b expression may get benefit from anti-IGF-IR mAb therapy; IGF-I/IGF-IR signaling may take advantage in tumor metastasis in these patients. In addition, Sorokin et.al reports that MEMO1 (mediator of ErbB2-driven cell motility 1) binds to insulin receptor substrate 1, activates the downstream PI3K/Akt signaling pathway, leads to up-regulation of Snail1 and thereby inducing the EMT program [140]. MEMO1 may act not only as a therapeutic target for cancer treatment but also as a potential biomarker for anti-IGF-IR therapy. Another team reports that reduction of CCN6 (WISP3) expression results in increased levels of IGF-I and activity of IGF-IR signaling pathway in mammary epithelial cells, which in turn is responsible for ZEB1-mediated EMT and invasion [141, 142]. Mutations in phosphoinositide-3-kinase, catalytic, alpha polypeptide (PIK3CA) may be associated with reduced sensitivity to IGF-IR/IR inhibitors [143]. Mucin 1 (MUC1), a transmembrane glycoprotein, as a critical downstream effector that mediates IGF-1-induced EMT in a PI3K/Akt signaling pathway-dependent manner in breast cancer [144]. Furthermore, survivin, a member of the inhibitor of apoptosis protein family, is also reported to be overexpressed in many tumor tissues. Activation of survivin by IGF-I signaling regulates IGF-I-induced EMT biomarkers and promotes migration ability in gastric cancer cells [145]. In addition, microRNAs have emerged as regulators in tumor metastasis by acting on multiple signaling pathways. Zhao et al. reported that microRNA-7 reversed EMT progression through targeting IGF-IR in gastric cancer [146]. All of these factors represent critical factors involved in IGF-IR-mediated EMT process, which may become potential biomarkers for identifying appropriate patients (Fig. 5). The potential biomarkers for anti-IGF-IR therapy that are involved in the regulation of EMT or have been indicated in clinical trials are listed in Table 2. Recently, our group has attempted to explore multiple classes of biomarkers including gene expression and mutations, which may carry greater predictive values on IGF-IR-associated tumor metastasis and survival. Future research is necessary to refine these biomarkers in preclinical studies and clinical trials on IGF-IR/IR inhibitors therapy.

**Table 2:** Biomarkers or potential candidates to IGF-IR inhibitory drugs

Biomarkers	Preclinical/clinical	Tumor type	Anti-IGF-IR strategy	Reference
EMT marker	Preclinical	Hepatocellular carcinoma	TKI Linsitinib	[8]
EMT marker	Clinical	Metastatic colorectal	Mab dalotuzumab	[127]
IR-A, IR-B, total IR	Clinical	Breast cancer	Mab cixutumumab	[108]
IRS1	Preclinical	Breast and colorectal cancer	Mab h10H5	[145]
IRS1	Preclinical	Breast cancer	TKI NVP-AEW541	[146]
IGF-I	Clinical	Metastatic colorectal	Mab dalotuzumab	[16]
PIK3CA	Preclinical	Different cancer cells	TKI Linsitinib	[136]
Potential biomarkers	Preclinical/clinical	Tumor type		Reference
Cbl-b	Preclinical	Gastric cancer		[32]
MEMO1	Preclinical	Breast cancer		[132]
CCN6 (WISP3)	Preclinical	Breast cancer		[133, 134]
Mucin 1	Preclinical	Breast cancer		[137]
MicroRNA-7	Preclinical	Gastric cancer		[139]

*Mab* monoclonal antibody, *TKI* tyrosine kinase inhibitor, *IR* insulin receptor, *IRS1* insulin receptor substrate 1, *IGF-I* insulin-like growth factor-I, *PIK3CA* phosphoinositide-3-kinase, catalytic, alpha polypeptide, *Cbl-b* E3 ubiquitin ligase Casitas B cell lymphoma-b, *MEMO1* mediator of ErbB2-driven cell motility 1, *MUC1* mucin1

#### Choose effective approaches to target pathway beyond the surface receptor

Since the IGF system comprises of multiple ligands and binding proteins, it has become evident that activation of other components of the IGF system may induce resistance to IGF-IR blocking therapies. The mechanism of resistance to specific IGF-IR inhibition therapy may be due to enhanced IR signaling, and co-targeting IGF-IR and IR signaling may acquire more response. Recently, the activity of an oral tyrosine kinase inhibitor (TKI) targeted IGF-IR/IR, KW-2450, was estimated in preclinical and phase I studies (NCT00921336). Four of 10 evaluable patients with advanced solid tumors showed stable disease. Single-agent was associated with modest antitumor activity and combination therapy needs further investigation in patients [147]. Huang, et al. reported that IRS-2 copy number gain, Kras and Braf mutation status were predictive biomarkers for response to the IGF-IR/IR inhibitor, BMS-754807 in colorectal cancer cell lines [148]. However, dual small-molecule TKI of the IGF-IR/IR used to exhibite undesirable outcomes in larger phase III trials [149]. Thus, more additional studies are necessary to determine whether these strategies can be translated into more clinical benefits.

Secondly, Insulin receptor substrate 1 (IRS1) is an adaptor protein that has the potential to transmit signals from IGF-IR proteins [150]. Activation of IGF-IR results in intrinsic tyrosine kinase phosphorylation and activates downstream adaptor protein IRS-1 and Shc, leading to activation of IRS-1/PI3K/Akt [84]. In addition to activation by IGF-IR, IRS1 has been reported to be stimulated by the growth hormone receptor and the ErbB family receptors independent of IGF-IR [151]. Preclinical data also shows that IRS1 promotes the induction of EMT process and cell proliferation in response to Wnt stimulation [152]. Components of IGF-IR signaling pathway such as IRS1 and IRS2 have been demonstrated to have predictive value in IGF-IR-targeting therapies in preclinical models of breast and colorectal cancer [153, 154]. Based on this data, it is reasonable to conclude that IRS1 may play a potential role in resistance to anti-IGF-IR therapy. However, more translational studies are necessary to determine whether patients with IRS1 overexpression who fail to respond to anti-IGF-IR therapy can get benefit from drugs targeting IRS1.

Thirdly, IGF-IR has extensive cross-talk with other receptor tyrosine kinases and their downstream factors, blocking of the IGF-IR signaling incompletely may be compensated by combination with other targeted therapy. Preclinical data has indicated that HER receptor signaling confers resistance to BMS-554417, an IGF-IR/IR inhibitor in both breast and ovarian cancer cells. Targeting HER-1 and HER-2 may overcome drug resistance to IGF-IR inhibitors [124]. Other researchers have investigated that treatment with combinatory IGF-IR and EGFR inhibitor therapy is synergistic in sarcoma and neuroblastoma cell lines [125]. Expression of IGF-IR predicts poor responses to EGFR TKI in NSCLC patients harboring activating EGFR mutations [14]. In addition to EGFR signaling pathway, some newly published data showed that co-targeting IGF-IR could sensitize triple-negative breast cancer to PI3K inhibition [155]. mTOR inhibitors are known to enhance IGF-IR signaling pathway leading to AKT downstream pathway activation [156]. The combination of IGF-IR inhibitor with mTOR inhibitors is currently being evaluated in clinical settings [157].

Finally yet importantly, since chemotherapy and radiation can induce IGF-IR activation and DNA repair mechanisms [158–160], combining IGF-IR targeted therapy to chemotherapy may be another potential effective strategy. It has been reported that IGF-IR TKI are capable of sensitizing wild-type and mutant BRAF melanoma cells to temozolomide [161]. Moreover, IGF-IR inhibition potentiates cytotoxic effects of chemotherapeutic agents in early stages of chemoresistant ovarian cancer cells [162]. Since these positive data are acquired from preclinical basic research, the feasibility and strategy of combining multiple targeted therapies and conventional cytotoxic medicine need to be further explored.

#### Suppressing cancer stem cell-like cells with over-activation of IGF-IR signaling

**Fig. 6:**
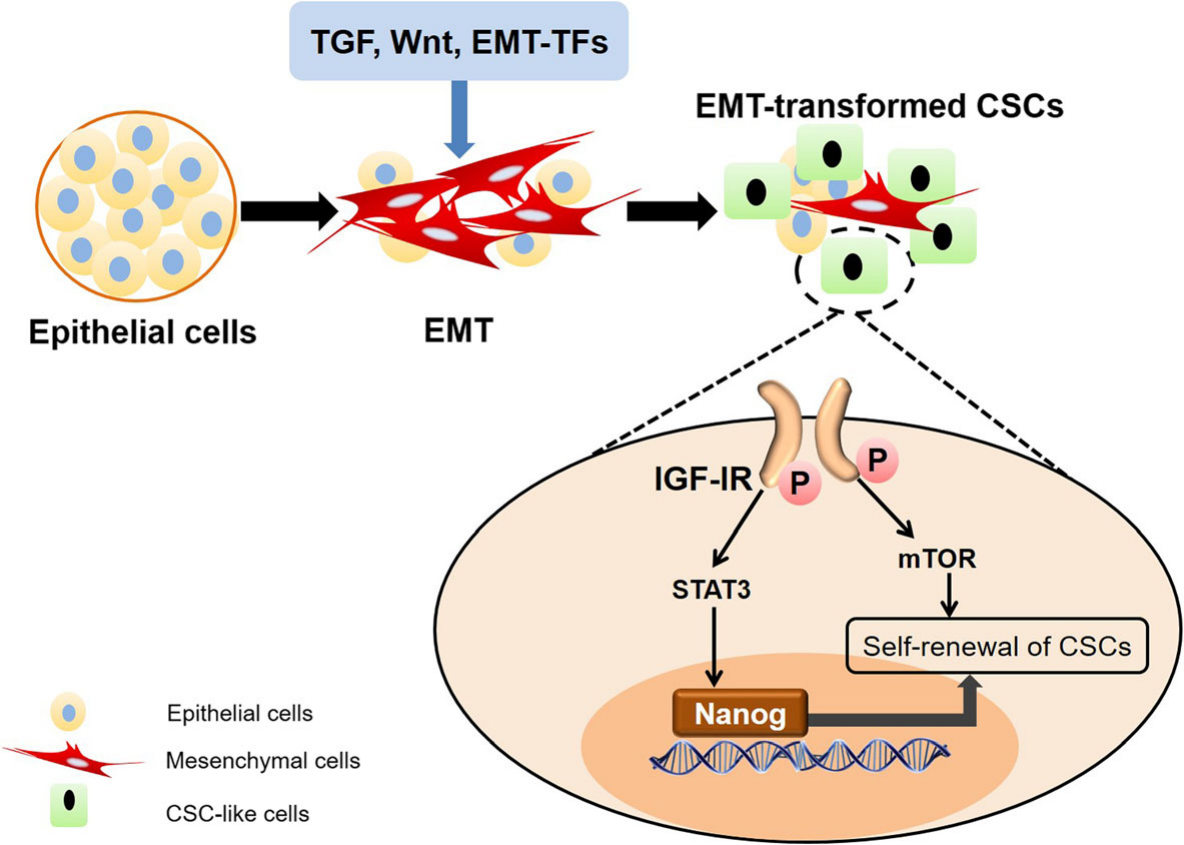
IGF-IR signaling in CSCs-like cells biology. Schematic summary of the IGF-IR signaling in the regulation of CSCs-like cells biology. After long term of EMT-associated factors effects, some of the EMT cells acquire CSCs-like properties with over-activation of IGF-IR signaling. IGF-IR/PI3K/Akt/mTOR signaling pathway activation increases the CSCs population, which promotes EMT process. The activation of IGF/STAT3/Nanog/Slug axis induces the progression of EMT and self-renewal of CSCs. CSCs, cancer stem cells; STAT3, signal transducer and activator of transcription 3; mTOR, mammalian target of rapamycin

Cancer stem cells (CSCs) are the other major contributor to tumor metastasis and drug resistance [49]. Recently, it has been observed that CSCs manifest EMT phenotype [163]; some of the EMT cells can acquire CSC-like properties which contributes to the metastasis and drug resistance [164]. For instance, overexpressing transcription factors of EMT, Snail and Twist, or under TGF-β exposure will induce stem cell features in non-tumorigenic human mammary embryonic cell [163, 165]. Disseminated breast cancer cells from pleural effusions are enriched with CSC-like population [166]. On the other hand, high expression of EMT markers are positively correlated with stem cell properties in colorectal and ovarian cancers [167, 168]. Therefore, suppressing CSC-like cells may be useful for inhibiting tumor metastasis and reversing multidrug resistance. Of note, IGF system has been demonstrated to play an important role in cancer progenitor/stem cells. Knockdown of IGF-IR or inhibition of its downstream pathway, PI3K/Akt/mTOR, can reduce the breast cancer stem cells populations and suppress EMT process in breast cancer cells [169]. Similarly, chemoresistant colon cancer cells exhibit CSC phenotype and hyperactive IGF-IR signaling. Treating this subtype of CSCs may enhance sensitivity to IGF-IR-targeted therapy [170]. Nanog is considered as a stemness maintainer and EMT facilitator. Yao. et al has reported that IGF/STAT3/Nanog/Slug axis induces the progression of EMT and self-renewal of CSCs, and may serve as potential therapeutic targets for colon cancer therapy [171]. Moreover, NANOG-positive CSCs isolated from hepatocellular carcinoma cells display higher levels of IGF-IR expression and exhibit resistance to therapeutic agents and high capacity for metastasis (Fig. 6) [172]. In summary, mounting evidence highlights the emerging role of IGF-IR signaling in cancer stem cell biology; IGF-IR can be considered as a marker of stemness. For the future development of anti-IGF-IR targeted therapy, it may be possible to produce specific inhibition agents targeted to CSC-like cells with over-activation of IGF-IR signaling.

## Conclusions

A growing body of evidence shows that the role of IGF-I/IGF-IR signaling is complex and multifactorial in the development and progression of tumor metastasis. Although data based on cellular and animal models have explored some mechanisms on IGF-I-induced EMT and tumor metastasis, complexity of cancer biology and heterogeneous of tumor bring a slew of setbacks for the IGF-IR-targeted therapies. The approach of treatment with the same drug to all patients and hoping for the best response seems unrealistic. In order to choose the optimal regime for each patient, we require a better understanding of which tumor is actually driven by IGF-I/IGF-IR signaling. This is equivalent to select advantage patients who can get benefit from anti-IGF-IR therapy according to predictive biomarkers. Therefore, it is necessary to explore more potential biomarkers via research on the mechanisms of IGF-I/IGF-IR regulating tumor metastasis and drug resistance. Hopefully, clinical trials involving anti-IGF-IR strategies will be designed with this principle in mind and more selected patients will get benefit from it.

## Abbreviations

Cbl-b: E3 ubiquitin ligase Casitas B cell lymphoma-b
EGFR: Epidermal growth factor receptor
EMT: Epithelial-mesenchymal transition
GSK-3β: Glycogen synthase kinase‐3 β
HER-2: Epidermal growth factor receptor 2
IGFBPs: Insulin-like growth factor binding proteins
IGF-IR: Insulin-like growth factor-I receptor
IR: Insulin receptor
MEMO1: Mediator of ErbB2-driven cell motility 1
MUC1: Mucin 1
NSCLC: Non-small cell lung cancer
PDGF: Platelet-derived growth factor
PIK3CA: Phosphoinositide-3-kinase catalytic, alpha polypeptide
RTKs: Receptor tyrosine kinases
TKI: Tyrosine kinase inhibitor
